# Who lacks and who benefits from diet diversity: evidence from (impact) profiling for children in Zimbabwe

**DOI:** 10.1186/s12942-020-00240-2

**Published:** 2020-11-04

**Authors:** Remco Oostendorp, Lia van Wesenbeeck, Ben Sonneveld, Precious Zikhali

**Affiliations:** 1 Vrije Universiteit Amsterdam, Tinbergen Institute, De Boelelaan 1105, 1081 HV Amsterdam, The Netherlands; 2 Amsterdam Centre for World Food Studies, Vrije Universiteit Amsterdam, De Boelelaan 1105, 1081 HV Amsterdam, The Netherlands; 3 World Bank, South Africa Office, The World Bank, 442 Rodericks Rd, Lynnwood, Pretoria, 0081 South Africa

**Keywords:** Child malnutrition, Zimbabwe, Impact assessment, Profiling

## Abstract

**Background:**

The impact of diet diversity—defined as the number of different foods or food groups consumed over a given reference period—on child nutrition outcomes strongly interacts with agro-ecological, institutional, and socio-economic drivers of child food and nutrition security. Yet, the literature on the impact of diet diversity typically estimates average treatment effects, largely ignoring impact heterogeneity among different groups.

**Methods:**

In this paper, we introduce a new method of profiling to identify groups of treatment units that stand to gain the most from a given intervention. We start from the ‘polling approach’ which provides a fully flexible (non-parametric) method to profile vulnerability patterns (patterns in ‘needs’) across highly heterogeneous environments [35]. Here we combine this polling methodology with matching techniques to identify ‘impact profiles’ showing how impact varies across non-parametric profiles. We use this method to explore the potential for improving child nutrition outcomes, in particular stunting, through targeted improvements in dietary diversity in a physically and socio-economically diverse country, namely Zimbabwe. Complex interaction effects with agro-ecological, institutional and socio-economic conditions are accounted for. Finally, we analyze whether targeting interventions at the neediest (as identified by the polling approach) will also create the largest benefits.

**Results:**

The dominant profile for stunted children is that they are young (6–12 months), live in poorer/poorest households, in rural areas characterized by significant sloping of the terrain and with one-sided emphasis on maize cultivation and medium dry conditions. When moving from “need” to “maximal impact”, we calculate both the coverage in “need” as well as the impact coverage, and find that targeting on need does not always provide the largest impact.

**Conclusions:**

Policy-makers need to remain alert that targeting on need is not always the same as targeting on impact. Estimation of heterogeneous treatment effects allows for more efficient targeting. It also enhances the external validity of the estimated impact findings, as the impact of child diet diversity on stunting depends on various agro-ecological variables, and policy-makers can relate these findings to areas outside our study area with similar agro-ecological conditions.

## Background

Zimbabwe continues to struggle with high levels of food and nutrition insecurity among its population, especially among children and women. In spite of the Millennium Development Goal (MDG) to reduce the proportion of malnourished children under 5, there was only a very limited decrease from 10 to 8 percent between 1999 and 2015 [28].^1^ As a result, the second Sustainable Development Goal to *end hunger, achieve food security and improved nutrition, and promote sustainable agriculture*, is one of the ten SDGs the government identified as areas of focus in 2016.

Zimbabwe’s continuing challenge to achieve food and nutrition security should be seen within its wider socio-economic, environmental and political context. From 2000 to 2009, the Zimbabwean economy collapsed in the face of severe macroeconomic imbalances and hyperinflation, when real Gross Domestic Product (GDP) per capita almost halved and while there has been economic growth since GDP per capita remains low at 1463 USD by 2019 with 34% of the people in extreme poverty.^2^ In the past decade, the country has suffered from recurrent droughts leading to severe food deficits, which not only caused immediate hardship and famine conditions, but also had a disproportionate negative impact on agro-based rural livelihoods. The negative impact on livelihoods was also widespread as smallholder farmers dominate the country’s agriculture as in 2017, close to 95% of agricultural households were considered smallholder agricultural households.

The recurrence of drought was further compounded by an uncertain agricultural policy environment following the government’s decision to abandon the willing seller-willing buyer approach to land reform and adopt the Fast Track Land Reform Programme (FTLRP) in 2000. The FTLRP was a radical land reform premised on extensive compulsory land acquisition and redistribution. The land redistribution program drastically decreased production of the four main commercial field crops—wheat, tobacco, soybeans, and sunflowers—due to low uptake and poor use of land, as well as the inexperience and lack of resources on the part of new farmers [18]. The impact on food security has been substantial and the main crops produced by smallholder farmers—maize, small grains, groundnuts, and cotton, among others—also recorded a decline in output.

In addition, evidence suggests that food aid and access to subsidized agricultural inputs has a history of being politicized in Zimbabwe. Duri and Amali [8] document the evolution of politicization of food aid, arguing that since attainment of independence in 1980, food aid has been used by the ruling party as an instrument of dominance, patronage, and subordination. In fact, rights-based NGOs such as the Zimbabwe Peace Project (ZPP) [31] and the Crisis in Zimbabwe Coalition have provided evidence that supports this phenomenon, with food aid being distributed on partisan lines to the benefit of mostly supporters of the ruling party—ZANU-PF. The Human Rights Watch identified politicization of emergency aid in the wake of tropical cyclone Idai in 2019.^3^ These are not isolated cases: ZPP has been continuously monitoring and documenting this practice since 2006 showing that the practice of distributing food and other aid in a partisan manner is prevalent and tends to increase towards elections. This means non-ruling party supporters are likely to be denied aid. In a context of high food insecurity, this has adverse implications on nutritional and dietary outcomes of the poor and vulnerable groups as they depend heavily on aid for survival.

Several generally accepted indicators can be used to measure the prevalence of household food and nutrition security status, including stunting (too short for age), wasting (too light for height) and underweight (too light for age). These robust indicators can be used to describe trends and patterns in child food and nutrition security in Zimbabwe, using the Zimbabwe Demographic and Health Surveys (DHSs) of 1988, 1994, 1999, 2005–2006, 2010–2011 and 2015.

Almost one in three (27 percent) children under the age of five in Zimbabwe were stunted in 2015, 3 percent wasted, 8 percent underweight and 6 percent overweight (too heavy for their age). Yet, the aggregate figures on child food and nutrition security in Zimbabwe hide persistent patterns at a more disaggregated level. For example, there are strong correlations of wasting and, especially, stunting with child age. The prevalence of stunting increases roughly until the age of 2.5 years and decreases afterwards.^4^ For moderate wasting, there is a peak at around 9–11 months, which seems to come later for severe wasting, with a declining trend after 1.5 years of age [6]. With respect to the gender dimension of child nutritional status, the literature is divided. While many studies conclude that boys have a more favorable nutritional status than girls [19, 25], consistent with a view that girls in many societies have a disadvantaged position, other evidence points at a more favorable nutritional status for girls [2], or that there is no significant difference [10]. For Zimbabwe, the data points at a lower nutritional status for boys than for girls, particularly for stunting [6].

**Fig. 1:**
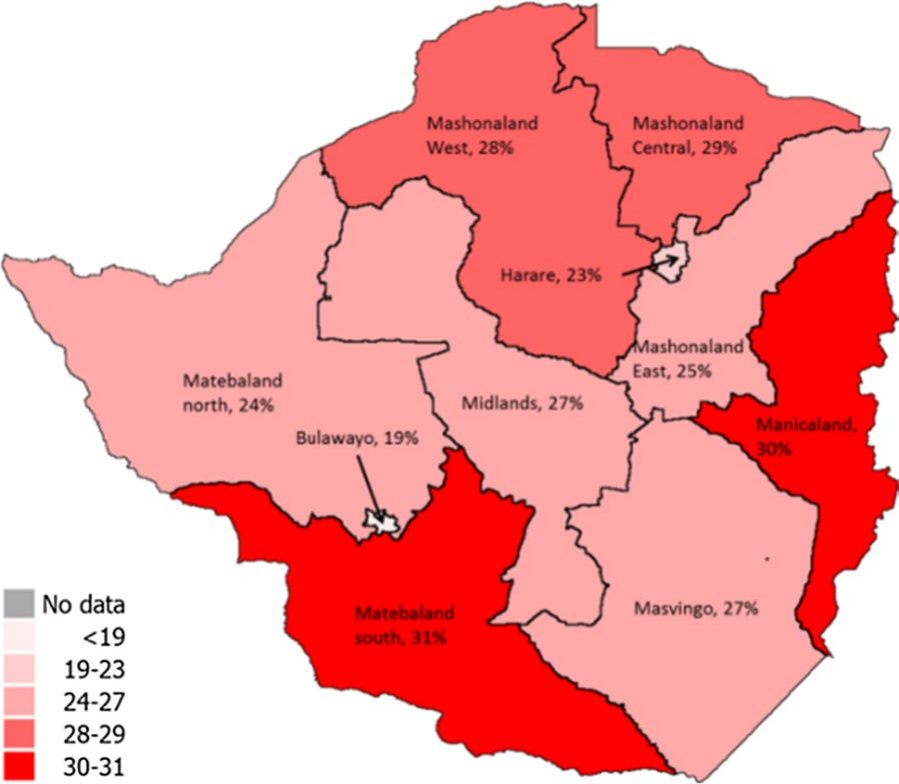
Percentage of children under five who are stunted, by province. Source: DHS 2015

One can also observe spatial patterns in child food and nutrition security (Fig. 1). Children in rural areas are more likely to be stunted than in urban areas (29 versus 22 percent in 2015) while the incidence of children who are overweight is higher in urban than in rural areas.^5^

In this paper the focus is on the link between nutrition outcomes and diet diversity, defined as the number of different foods or food groups consumed over a given reference period. The World Health Organization uses the number of food groups a child is fed as a proxy for dietary diversity on the premise that it gives an indication of adequate micronutrient-density of foods. Speficifically, feeding the child food from at least four food groups is set as a minimum requirement for dietary diversity. The Zimbabwe DH 2015 reveals that close to a third of children (28.3 percent) aged 6 to 23 months consumed at least four food groups, showing adequate diet diversity. The proportion is less in rural (21.6 percent) than in urban (46 percent) areas, while, children in poor households also tend to be less likely to consume vegetables and tubers which negatively affects their nutritional status (Poverty and Poverty Datum Line Report 2011/12).

The descriptive evidence clearly shows that Zimbabwe faces severe nutritional challenges. Improving diet diversity is therefore an important policy objective, as nutritional deficiencies have been known to impact heavily on the mental and physical development of children, such as increasing the probability of stunting (e.g. see [14, 16, 21], among many others). Improvements in diet diversity should also take into account local conditions, as the relationship between diet diversity and child nutrition outcomes has been shown to be heterogeneous (e.g. [1, 27]. This policy imperative raises therefore two issues. First, who exactly lacks diet diversity, when moving beyond simple bivariate analyses? Second, who benefits most from increased diet diversity, considering the complexity of the diet diversity-nutritional outcome nexus?^6^

This paper focuses on the link between diet diversity and stunting, and it can be argued that absolute caloric intake should also be taken into consideration. However, there is a large body of literature specifically linking stunting to *diet diversity* rather than energy deficiencies (e.g. [1, 5, 7, 17, 20, 22, 25, 26]). Also, it is hard to conceive of interventions that would improve diet diversity but would decrease energy intake, this would imply that staple foods are *replaced* by low-calorie, but high-mineral and vitamin food sources.^7^

In the next section we discuss two methodologies to identify, respectively, the diet diversity deficient group, and who benefits most from increasing diet diversity. First, we review a fully nonparametric ‘polling approach’ introduced by Wesenbeeck et al. [29] which can be applied to profile households with young children suffering from inadequate diet diversity. Second, we discuss an extension of this profiling methodology by combining it with matching techniques to identify ‘impact profiles’. These profiles are nonparametric groups of treatment units, c.q. households with young children, combined with an estimated *impact* of enhancing diet diversity on child stunting.

Studies using impact evaluation techniques fail to capture complex impact heterogeneity. This is not on purpose but a consequence of the fundamental fact that one cannot observe the same person in a programme and out of it [11]. The main idea of our impact profiling method is to use theoretically grounded empirical analysis to identify non-parametrically the subgroups with the largest impact, by combining the concept of profiling (and polling approach) with impact evaluation (matching) techniques.^8^ The largest impact will be found for subgroups which have a large population, which include many potential beneficiaries, and where the average treatment effects on the treated are large.

Before introducing the method, we raise an important caveat. Given that our proposed impact profiling approach combines polling and matching methodologies, it automatically invokes the latter’s assumption of selection on observables. To the extent that matching does not fully control for all confounders, one should be cautious in making causal inferences also with our method. Nevertheless, our approach allows for the systematic identification of potentially important heterogeneous ‘associations’, irrespectively whether one is willing to interpret them as (entirely) ‘causal’ in any given empirical application.

## Methods

### The polling methodology

A fully nonparametric profiling approach can be found in the ‘polling’ methodology introduced by Wesenbeeck et al. [29]. Here we provide an intuitive explanation (see Box 1 for a formal exposition of the polling methodology). Suppose that observed diet diversity is linked to a household *profile*, i.e. That different variables are evaluated jointly with diet diversity to assess the likelihood of being associated with adequate dietary diversity. Formally, observed values of the variables used in the analysis define a joint empirical frequency distribution. Conditional frequency distributions can be derived from this joint distribution by partitioning the answers by, say, $S$ respondents indexed $s$ into a dependent variable $y$ (c.q. diet diversity with values ‘adequate’ and ‘inadequate’) and a vector $x$ of independent variables (‘profile’ $x$), taking the frequencies of $y$ conditional on $x$.^9^ The conditional frequencies are naturally interpreted as probability estimates of $y$ given profile $x$. The *coverage* of a profile $x$ is the mass of the respondents within profile $x$ in the relevant group (c.q. with inadequate diet diversity) divided by the total mass of respondents in the relevant group. In empirical applications, a balance needs to be struck between including a relatively large set of variables in the profile, with a high degree of specificity, but a low number of observations in each profile, or a small set, with a broad coverage, but less specificity.

In analogy with errors of the first and second kind, two types of errors can be identified in polling analysis. First, an *exclusion error* ${\lambda }_{polling}$ of profile $x$ is defined as the total mass of the relevant group not covered by a profile $x$ (i.e. one minus the coverage). Secondly, an *inclusion error* ${\mu }_{polling}$ is the total mass of households included in a profile $x$ while the household does not belong to the relevant group (i.e. in our case, experiences adequate rather than inadequate diet diversity). Selection of a “winning” polling profile can be based on a minimization of exclusion and inclusion errors, weighted to yield one indicator ${\psi }_{polling}$. Here we use a weighted geometric mean of errors with weight $\alpha$ on the exclusion error and weight $1-\alpha$ on the inclusion error ($0\le \alpha \le 1$).

An alternative to profiling is to estimate a binary response model (such as probit or logit) to estimate the probability that the dependent variable $y$ takes on a given value as a function of the vector of values of the independent variables $x$ (e.g. $Prob\left[\mathrm{inadequate\ diet\ diversity}|x\right]=F({x}^{^{\prime}}\beta$)). This approach identifies correlates of outcomes of $y$ with value of $x$, but it does not take into account the probability density of $x$ ( ‘mass’) and therefore also not the inclusion and exclusion errors. For instance, the binary response model may be able to identify how likely it is that someone is poor who lacks a job, has little education, etc., but it will be silent on how many people are in this class and how many are not covered (‘exclusion error’) and how many are actually non-poor (‘inclusion error’).^10^ Moreover, the binary response model is a fully parametric approach relying on a specific functional form $F$ (e.g. logistic distribution in the case of a logit model). Polling is actually a simple approach to profiling that takes into account inclusion and exclusion errors without imposing unnecessary parametric constraints.

Box 1. The polling approach ${c}_{s}=\left({c}_{1s}, \dots ,{c}_{rs},\dots ,{c}_{Rs}\right): \mathrm{value\ of }\,{x}_{s} (\mathrm{integer\,coded})$

$${g}_{s}: \mathrm{value \,of\, }{y}_{s} (\mathrm{integer \,coded})$$

$${w}_{s}: \mathrm{mass \,of\, the \,observation\ }s,\mathrm{ with }\,s=1\dots S$$

$${S}_{gc}: \mathrm{set \,of \,observations\, with }\,{y}_{s}=g, {x}_{s}=c$$

$$\mathrm{mass\ of\ }{S}_{gc}: {m}_{gc}=\sum_{s\in {S}_{gc}}{w}_{s}, {\text{ coverage}}:\frac{{m_{{gc}} }}{{\mathop \sum \nolimits_{c} m_{{gc}} }}$$

$$\mathrm{exclusion\, error}: {\uplambda }_{polling}=1-\frac{{m}_{gc}}{\sum_{c}{m}_{gc}} (1-\mathrm{coverage})$$

$$\mathrm{inclusion\,error}: {\upmu }_{polling}=1-\frac{{m}_{gc}}{\sum_{g}{m}_{gc}}$$

$$\mathrm{indicator}: {\psi }_{polling}={\lambda }_{polling}^{\alpha }{\mu }_{polling}^{1-\alpha }$$

Source (with adaptation): Wesenbeeck et al. [29].

### The impact profiling methodology

The value of a profiling (and therefore also polling) approach is to identify the households that are most in ‘need’ of diversification of their small children’s diets. The polling approach is additionally valuable because it provides a fully non-parametric approach to profiling, allowing for the highly complex nature of the agriculture-food-nutrition nexus, involving a manifold of intermediating agro-ecological, institutional and socio-economic factors [24].

Targeting of interventions to enhance diet diversity based on profiling assumes that the impact of the intervention varies by some measure of need, c.q. that children with the lowest diet diversity will benefit the most from an intervention to increase diversity. This is however an untested assumption, and there is no reason to assume this to hold, for at least two different reasons, namely measurement and interaction effects. First, the measure of dietary diversity may hide underlying variations in the composition of the diet as the same score can be achieved by different combinations of food groups. As a result, the same level of diet diversity (i.e. representing similar ‘need’) may result in different impacts when contextual factors are related to the underlying composition, and therefore nutritional value, of the diet.^11^ Secondly, although dietary diversity is an important factor in determining stunting, there will be interaction effects with other factors such as the prevalence of diseases (diarrhea) and parasites. Locational factors including sanitary practices and access to clean water may therefore affect the impact of otherwise identical diets.

It is possible, however, to combine the polling approach with the impact evaluation approach in order to identify groups of treatment units that stand to gain the most from a given intervention. This new approach of impact profiling combines the strengths of both approaches, namely rigorous impact analysis but allowing for multidimensional and non-parametric interactions affecting the impact pathway.

Using the same notation as for the polling approach above, let ${x}_{s}$ denote a vector of independent variables that interact with the impact of interventions affecting dietary diversity on child nutrition outcomes for households $s=1,\dots S$.^12^ Let $ATT(x)$ measure the estimated average treatment effect on the treated of a change from adequate to inadequate dietary diversity on a nutrition outcome for children in households with ‘profile’ $x$, i.e. the set of households $s\in {S}_{x}\subset \{1,\dots ,S\}$ with characteristics ${x}_{s}=x$.^13^ As in the polling approach, a balance should be struck between including a relatively large set of variables in the profile, with a high degree of specificity, but a low number of observations in each profile (and less precise impact estimates), or a small set, with a broad coverage (and more precise impact estimates), but less specificity. This does not mean that the full set of variables included in ${x}_{s}$ cannot be covered, but only that in empirical applications often not all conceivable interactions can be included simultaneously.

The average treatment effect on the treated $ATT(x)$ can be estimated by a matching technique, such as propensity score matching. The variables *x* are included among the propensity score matching variables as they are both assumed to be related to the treatment as well as the outcome. For the case of improving dietary diversity, the $ATT(x)$ is then calculated by matching households with young children and with inadequate dietary diversity within profile *x* (the treatment group) with ‘similar’ households enjoying adequate diversity. This gives the impact of the improvement in dietary diversity on child nutrition outcomes for children with profile *x* and inadequate dietary diversity, i.e. the $ATT(x)$. The $ATE(x)$ is then given by multiplying $ATT(x)$ by the proportion of young children experiencing inadequate dietary diversity within profile *x*, $Pr[\mathrm{inadequate\ dietary\ diversity}|x]$.

For the final step of the impact profiling method, we note that the size of the population of profile $x$ is given by $\mathrm{Pr}\left[x\right]=\sum_{s\in {\mathrm{S}}_{x}}{w}_{s}$. The share of treated within a given profile $x$ is given by $Pr[\mathrm{inadequate\ dietary\ diversity}|x]$, i.e. the share of households within profile $x$ with inadequate diet diversity among the young children. The impact of a change in diet diversity from adequate to inadequate among households within profile $x$ is then given by

1 $$\mathrm{Impact \,profile\, }x= ATT(x)\times Pr[\mathrm{inadequate \,dietary \,diversity}|x]\times \mathrm{Pr}[x]$$

Equation (1) shows that targeted interventions to improve diet diversity should look at profiles that have a large population (large $\mathrm{Pr}[x]$), which include many potential beneficiaries (large $Pr[\mathrm{inadequate\ dietary\ diversity}|x]$), and where the average treatment effects on the treated ($ATT(x)$) are large.^14^ Analogously to the polling approach, we define the *impact coverage* of a profile $x$ as the ratio of the impact on households with profile $x$ to the total impact across all possible profiles (${x}^{^{\prime}}$):

2 $$\mathrm{Impact\,coverage}=\frac{ATT(x)\times Pr[\mathrm{inadequate\, dietary \,diversity}|x]\times \mathrm{Pr}[x]}{\sum_{{x}^{^{\prime}}}ATT({x}^{^{\prime}})\times Pr[\mathrm{inadequate\, dietary \,diversity}|{x}^{^{\prime}}]\times \mathrm{Pr}[{x}^{^{\prime}}]}$$

Note, however, that Eq. (2) assumes that the total impact across all profiles (the denominator) is the same as the total impact across all households. This is not correct if there are overlapping profiles, i.e. some households may be included in more than one profile. In this case we can calculate the total impact by adjusting the denominator in Eq. (2):

3 $$\sum_{{x}^{\mathrm{^{\prime}}}}ATT({x}^{\mathrm{^{\prime}}})\times Pr[\mathrm{inadequate\, dietary\, diversity}|{x}^{\mathrm{^{\prime}}}]\times \overline{\mathrm{Pr}}[{x}^{\mathrm{^{\prime}}}]$$

where $\overline{\mathrm{Pr}}\left[{x}^{\mathrm{^{\prime}}}\right]=\sum_{s\in {S}_{{x}^{\mathrm{^{\prime}}}}}{w}_{s}/{c}_{s}$ and ${\mathrm{c}}_{\mathrm{s}}$ is the number of times that household s is included across all possible profiles.

We define the impact exclusion error ${\lambda }_{impact}$ as the complement of the impact coverage, i.e.

4 $${\lambda }_{impact}=1-\mathrm{impact\, coverage }$$

The inclusion error ${\mu }_{impact}$ is defined as in the polling approach as the total mass of households with already adequate diet diversity that are included in profile $x$ and hence which do not benefit from an intervention to change inadequate into adequate nutrition:

5 $${\mu }_{impact}=1-Pr\left[\mathrm{inadequate\,dietary\, diversity}|x\right]$$

The wining impact profile ${x}^{*}$ is the profile that minimizes both the impact exclusion and inclusion errors ${\lambda }_{impact}$ and ${\mu }_{impact}$. Because there might be a trade-off between these two errors, we define the weighted geometric mean of errors impact indicator function ${\psi }_{impact}={\lambda }_{impact}^{\alpha }{\mu }_{impact}^{1-\alpha }$, where $0\le \alpha \le 1$ reflects the relative weight put on the exclusion error (compared to the inclusion error). The winning impact profile ${x}^{*}$ is then given by ${x}^{*}={argmin}_{x}\left\{{\lambda }_{impact}^{\alpha }{\mu }_{impact}^{1-\alpha }\right\}$. Interventions should be targeted at winning impact profiles, given that they tend to have large coverage and less inclusion error.^15^

### Identification of variables

For the construction of the dependent variable *y*, we use information on food groups consumed as reported in the DHS 2015 survey. Specifically, we construct a Child Dietary Diversity Score (CDDS) using the methodology for children’s nutrition described in Swindale and Bilinsky [26]. Instead of the commonly used 12 food groups, 8 are used in a 24 h recall question (‘have you given your child [ITEM] yesterday?’) with possible answer ‘yes’/’no’.^16^ These 8 groups are: (1) Grains, roots and tubers, (2) Vitamin A rich foods (orange fruits and vegetables), (3) Other fruits and vegetables, (4) Meat, poultry, fish and seafood, (5) Eggs, (6) Pulses, legumes, nuts, (7) Milk and milk products, and (8) Foods cooked in oil or fat.

In the DHS data for Zimbabwe, observations are available for 13 food items or groups. The mapping from those items to the first 7 of these 8 food groups is summarized in Table 1. We note that there is no measurement of food being cooked in oil or fat. Aggregation of classes implies that a score of 1 is assigned whenever at least one of the original classes scores a 1. Applying this method leads to a CDDS with a potential score from 0–7, that needs interpretation in terms of sufficiency of the dietary diversity. Following the DHS standards, we define a consumption of more than 3 food groups as adequately diverse, and equal or below this as inadequately diverse.

**Table 1:** Mapping from DHS food categories to CDDS classes

DHS category	CDDS class
Bread/noodles	Grains, roots and tubers (1)
Potato, cassava	Grains, roots and tubers (1)
Eggs	Eggs (5)
Meat	Meat, poultry, fish and seafood (4)
Orange vegetables	Vitamin A rich foods (2)
Dark green leafy vegetables	Other fruits and vegetables (3)
Orange fruits	Vitamin A rich foods (2)
Other fruits	Other fruits and vegetables (3)
Organ meat	Meat, poultry, fish and seafood (4)
Fish, shellfish	Meat, poultry, fish and seafood (4)
Beans, peas, lentils	Pulses, legumes and nuts(6)
Milk and milk products	Milk and milk products (7)
Insects	Meat, poultry, fish and seafood (4)

The DHS 2015 survey asks about the diet for children born in 2013–2015, c.q. children 0–25 months old. In case there are multiple children in this age group, the diet of the youngest child is being asked. Since children below the age of 6 months presumably are bottle- or breastfed, we exclude these children from the analysis of diet diversity.

The DHS survey is a cross-sectional data set measuring diet diversity in the past 24 h at the time of the household interview. Because the survey was implemented during the 5-month period of July-December, we corrected for the artificial temporal pattern in measured diet diversity due to nonrandom interview timing (see Additional file 1).

By combining the socio-economic dimension with the biophysical environment, this study arrives at a comprehensive profile of households suffering from inadequate child dietary diversity. For this, data on agronomic and ecological characteristics need to be linked to the DHS households. However, the geographical link between DHS data points and gridded attribute maps could not be established by a straightforward crossing exercise. The reason is that rasterization of point data retains one single DHS observation per grid. Hence, a stepwise spatial protocol was used.^17^

A total of 18 variables were identified (see Additional file 3 for definitions and descriptive statistics). Next we ran univariate regressions of the dependent variable (adequate diet diversity) on each of these variables and selected the top-10 variables in terms of lowest p-value (see Additional file 3: Table S.3.3).^18^ This generated the following list of “promising” variables for inclusion in the polling analysis: age of the child, child has sibling(s) of up to 5 years (child characteristics), working status of mother, education of mother, location, wealth quintile (household characteristics), and land use, slope of terrain, length of growing period, and farming system (biophysical characteristics).

## Results

In this section we apply the polling approach to profile households with small children suffering from inadequate diet diversity in Zimbabwe in 2015. This analysis will answer the question who is lacking in diet diversity. We also apply our extension of the polling approach, the impact profiling approach, to profile households in which small children would benefit the most from increased diet diversity through reduced stunting. This will answer the question who will be benefitting the most from increased diet diversity. And, finally, we compare both approaches to answer the question whether targeting the neediest (as identified in the polling approach) will also create the largest benefits (as in the impact profiling approach). We will show that need and impact are not the same and this divergence suggests that there are other binding constraints that need to be overcome apart from (measured) inadequate diet diversity.

We limit the analysis to the most recent DHS (rather than using multiple waves) for two reasons. First, we study a highly context-dependent relationship between diet diversity and stunting (as confirmed by the results below). This relationship is unlikely to remain the same over time, especially in the case of Zimbabwe which has been suffering from a relentless sequence of deep crises (droughts, hyperinflation, land reform issues, political upheaval). Second, the use of multiple waves would indeed be highly beneficial if it would allow for the inclusion of, say, household or village fixed effects to control for time-invariant unobservables. Unfortunately, this is not feasible given that the different DHS waves are based on different samples over time and therefore not a panel.^19^

### Who lacks diet diversity? results from the polling approach

Earlier research [29] has shown that the best combination of inclusion and exclusion errors of profiles across outcome categories is obtained when using 5 variables in the potential profile for polling.^20^ Since the number of observations for children between 6 and 25 months is rather limited (1613), we report results at the national level only, using the geo-specific indicators to allow for a representation of the spatial heterogeneity emphasized in section 1. The use of 10 explanatory variables in groups of 5 variables leads to a total number of possible combinations of 252. Depending on the weighing of the exclusion and inclusion error (i.e. parameter $\alpha$ in Box 1), where a higher value of $\alpha$ corresponds to shifting attention away from the inclusion to the exclusion error, a different “winning” profile results.

Additional file 6: Table S.6.1 summarizes the winning profiles for the different ranges of α (using steps of 0.1), with statistics for exclusion and inclusion error and indicator score, and adds some notes on interpretation. The selection of variables depends on the choice of alphas. For instance, winning profiles include children of ages between 6–12 months for high values of α (i.e. emphasis on the exclusion error), while for lower values of α (i.e. emphasis on avoiding inclusion errors), well-known correlations with wealth and working status of the mother appear. For all values of α, locational variables matter: rural households in medium length growing periods are in all winning profiles, while light/moderate slopes and maize/mixed farming system figure for higher values of α and high shares of grass and woodland for lower values. Jointly, the location variables point at areas where agriculture is challenged by a variety of natural conditions.

### Who benefits most from moving from inadequate to adequate diet diversity? Results from the impact profiling approach

The above profiling (polling) analysis suggests that interventions for improving diet diversity among children ages 6–25 months should be targeted, in the first instance, at rural areas with light/moderate slopes (8–30%), medium length of growing period (121–180 days), maize/mixed farming system, and young children between the age of 6 and 12 months living in the poorest/poorer households with a non-working mother.

We now redo this analysis using the impact profiling methodology introduced in Sect. 3, using the same dataset, concepts, and variable definitions, to identify household profiles for which the largest *impact* of improving diet diversity on small children can be expected.^21^ This will also allow a comparison between the outcomes of the polling and impact profiling approaches for our study case of child diet diversity and stunting.

The empirical implementation of the method of impact profiling requires an estimate of the ATT. Because we are interested in the impact of improving diet diversity on households which *currently suffer from inadequate child diet diversity*, we define treatment as a change from adequate to inadequate child diet diversity. As outcome variable we take stunting, defined as a height-for-age score more than 2 standard deviations below the mean score.

In terms of independent variables, we will use the same variables as used in the polling analysis for two reasons. First, many of these variables that are linked with the incidence of diet diversity are also related to its nutritional impact with many parents trying to optimally choose (c.q. increase) diet diversity to improve the nutrition of their children. Hence, these variables are exactly the variables that should be included in a matching estimator as they are both affecting the outcome and treatment variables.^22^ Secondly, we choose the same variables because we like to investigate to which extent a focus on diet diversity needs overlaps with a focus on diet diversity impact.

We note that we do not control for calorie intake which may be related to diet diversity as well as stunting.^23^ The DHS data do not include observations on calorie intake and hence we are, effectively, taking a semi-reduced form approach, where the latent calorie intake variable is controlled for by the exogenous variables. Therefore, the various control variables in the analysis, such as age of child and wealth status of household should be interpreted as affecting stunting directly as well as indirectly through calorie intake.^24^

There are different approaches to estimating the ATT, and for our application we will use the propensity score matching method which has become a popular approach to estimate causal treatment effects, as it provides a solution to the fundamental evaluation problem that we only observe households that are treated or untreated, but not both [23].

The next step in our analysis is therefore the specification of the propensity score model. As pretreatment variables we can in principle include all the variables that were listed in (Additional file 3: Table S.3.1). When we estimate the propensity score using a probit specification, we find that the resulting propensity scores do not create a balance of each of the covariates across treatment and comparison groups. This is also the case if we add regional dummies. This means that the propensity score model needs to be modified, for instance by dropping variables that are less theoretically important, recategorizing variables, including interactions between variables, or including higher-order terms or splines of variables [9].

For the polling analysis we selected the top-10 variables (Additional file 3) in terms of statistical significance when explaining diet diversity. These variables are strongest related to whether child diet diversity is inadequate in a household, i.e. whether the household is selected into treatment, and therefore, we re-estimated the propensity score model with these variables. Also balance in covariates across treatment and comparison groups was achieved (see below).

The estimated propensity score model (Table 2) shows that the probability of inadequate diet diversity decreases with the age of the child but at a decreasing rate (the marginal effect becomes zero at around 2.2 years). Children who have at least one sibling of age 5 years or younger have a 5% point higher probability of suffering from inadequate diet diversity. There is also a strong negative relation with education and especially children with mothers without any education are much more likely (at least 25% point) to suffer from inadequate diet diversity than children with mothers with higher levels of education. There is also a negative and relatively monotonous relationship with household wealth, with children in the poorest quintile having a 16–17% point higher probability of suffering from diet diversity than children in the two highest wealth quintiles. Children in urban households have a 11% point lower probability of suffering from inadequate diet diversity compared to similar rural households. Finally, children living in areas where more than 50% of land is cultivated have a much higher chance of suffering from diet diversity.

**Table 2:** Probit estimates for being enlisted in the intervention (marginal effects)

	Estimate	s.e
Age of child (years)	− 0.97***	0.20
Age of child squared	0.22***	0.08
Child has sibling(s) of ≤ 5 years (dummy)	0.05**	0.03
Mother is working in the last 7 days (dummy)	− 0.09***	0.03
Education of mother (omitted category: ‘No education’)		
Primary (dummy)	− 0.25*	0.14
Secondary (dummy)	− 0.26*	0.14
Tertiary (dummy)	− 0.33**	0.13
Wealth quintile (omitted category: ‘Bottom quintile’)		
2nd quintile (dummy)	− 0.06	0.04
3rd quintile (dummy)	− 0.10**	0.04
4th quintile (dummy)	− 0.17***	0.04
5th quintile (dummy)	− 0.16***	0.04
Urban (dummy)	− 0.11***	0.03
Land use (omitted category: ‘ > 50% cultivated land’)		
> 50% forest/barren land (dummy)	− 0.26***	0.08
> 50% grass and wood land (dummy)	− 0.21***	0.08
> 50% built up land (dummy)	− 0.25***	0.08
Land cover associations (dummy)	− 0.26***	0.08
Length of growing period (omitted category: ‘0–75 days’)		
76–120 days (dummy)	− 0.03	0.07
121–180 days (dummy)	− 0.04	0.08
> 180 days (dummy)	0.02	0.11
Farming systems (omitted category: ‘Highland temperate mixed’)		
Root crop/Cereal-root crop mixed (dummy)	0.14	0.13
Maize mixed (dummy)	0.01	0.10
Large commercial and smallholder/Pastor (dummy)	0.22*	0.14
Agropastoral millet sorghum (dummy)	0.05	0.12
Slope 8–30 degrees (dummy)	− 0.02	0.05
Slope > 30 degrees (dummy)	− 0.08	0.07
N	1613	

Dependent variable: inadequate diet diversity

Marginal effects evaluated at the mean values. Inference: ^***^ p < 0.01; ^**^ p < 0.05; ^*^ p < 0.10. Coefficients significant at 10% are in italic

We verified that there is sufficient overlap in the range of propensity scores across treatment and comparison groups, i.e. in common support, except at the very upper end of the propensity score distribution where 1.1% of the treated observations fall outside (see Additional file 4: Figure S.4.1). Overall, therefore, there is sufficient common support for propensity score matching. Comparing Additional file 6: Table S.6.1 and Table 2, shows that there is considerable overlap between the statistically significant variables in the propensity score model (Table 2) and the variables selected for the winning profiles (Additional file 6: Table S.6.1). Nevertheless, there are also noticeable differences with the length of growing period and slope only featuring in the winning profiles. These differences arise as a binary response model is fully parametric as well as not taking into account the probability density of $x$ (‘mass’) and therefore the inclusion and exclusion errors, unlike the polling approach.^25^

The propensity score method assumes that there is ‘balancing’ in the sense that the propensity score and covariates should have similar distributions in the treated and comparison groups [13]. Here we choose the widely used kernel matching method where comparison households on the common support are weighted by their distance in propensity score from treated households (and all households outside the support are omitted). The type of kernel applied is the Epanechnikov kernel.

**Fig. 2:**
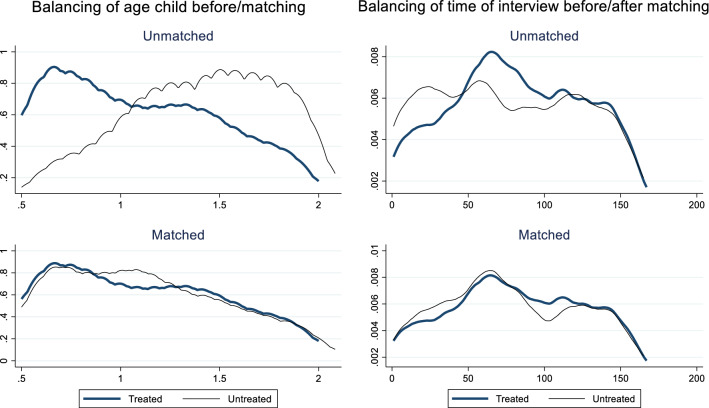
Density plots of mean age of child and time of interview, before and after matching

The results of different balancing tests suggest that our constructed comparison groups are well-balanced (see Additional file 4). As an illustration, Fig. 2 below shows that the age profile of the child becomes highly comparable after matching. The same holds true for the day that the interview was conducted, alleviating concerns that seasonal variations are confounding the results.

The next table presents the average treatment effect on the treated. While there is a statistically significant (and unexpectedly) negative difference in child stunting between the households without (treated) and with (comparison) child diet diversity (difference − 0.05, *t* = − 2.50), there is no longer a significant difference after controlling for observable differences between these two groups, i.e. after matching (difference − 0.01, t-value − 0.22).^26^

**Table 3:** Estimated average treatment effect on the treated (ATT) of inadequate diet diversity on stunting, children ages 2 and below

	Treated	Comparison	Difference	s.e	t
Unmatched	0.20	0.26	− 0.05	0.02	− 2.50
ATT	0.21	0.22	− 0.01	0.03	− 0.22

Approximate standard errors are calculated for the treatment effects assuming independent observations, fixed weights, homoscedasticity of the outcome variable within the treated and within the control groups and that the variance of the outcome does not depend on the propensity score [15]

The fact that the overall ATT is not significantly different from zero suggests two possible interpretations.^27^ First, there is no relationship between child diet diversity and stunting in Zimbabwe. This is, however, unlikely to be the case, as the relationship between diet diversity and stunting has been observed in many instances and there is no reason to believe that a similar mechanism is not operating in Zimbabwe. A second interpretation is that there is a relationship between diet diversity and stunting in Zimbabwe, but the strength of the relationship varies depending on contextual factors reflecting the highly complex nature of the agriculture-food-nutrition nexus involving a manifold of intermediating agro-ecological, institutional and socio-economic factors.

**Fig. 3:**
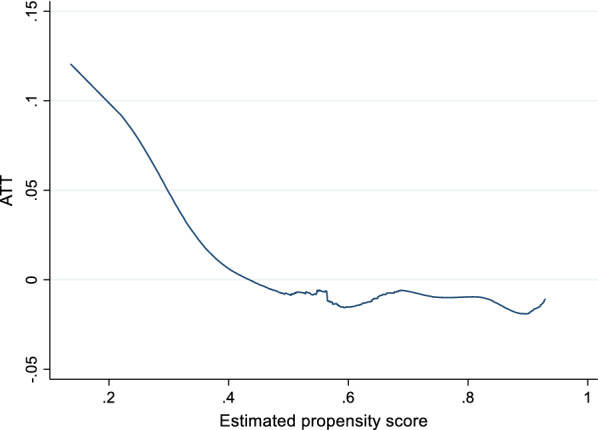
Average Treatment Effect on the Treated by estimated propensity score

If the second interpretation is the correct one, we would expect significant average treatment on the treatment effects for subsamples of the population. Impact heterogeneity is also the key assumption underlying targeting on the neediest, assuming that impact increases in need. In order to test for this possibility, we show a locally weighted regression of the difference in stunting between treatment and comparison observations across estimated propensity scores for having inadequate diet diversity in Fig. 3. Targeting on the needy would suggest that this regression would be upward sloping throughout. This is not the case, however, as the average treatment effect on the treated falls throughout. This is suggestive evidence that targeting on households with the highest likelihood of suffering from inadequate diet diversity is unlikely to be the most effective approach when reducing stunting.

This evidence on the need for targeting on impact rather than need is suggestive but not yet conclusive as Fig. 3 does not take into account probability densities (‘mass’) and inclusion/exclusion errors. We therefore continue with the impact profiling approach which does take into account probability densities, inclusion/exclusion errors, and which is nonparametric additionally. Similar to the polling application, we iterate through all possible combinations of values for any subset of 5 variables of the top-10 variables listed in (Additional file 3: Table S.3.3). For each of these combinations (‘profiles’), we calculate the average treatment on the treated effect using the estimated propensity scores from the regression reported in Table 2.

At this point it is important to discuss a possible objection to the impact profiling approach. One may argue that the estimation of treatment effects for subgroups is vulnerable to turning into a ‘fishing expedition’. Indeed, even if the treatment effect is zero for all treatment units, then it is highly likely that one will find nonzero treatment effects for some subsamples simply because of sampling variation. However, there are three ways to test whether estimated nonzero treatment effects for subsamples reflect actual nonzero treatment effects. First, if nonzero treatment effects are merely reflecting sampling variation, then the distribution of estimated treatment effects will be centered around zero. Second, the central tendency of the distribution of treatment effects should be in the direction predicted by theory and previous evidence. Third, in case the treatment effect is nonnegative (nonpositive), the distribution of estimated treatment effects will shift towards the right (left) with increasing sample size and at large sample sizes all estimated treatment effects that are statistically significant will be positive (negative).

**Fig. 4:**
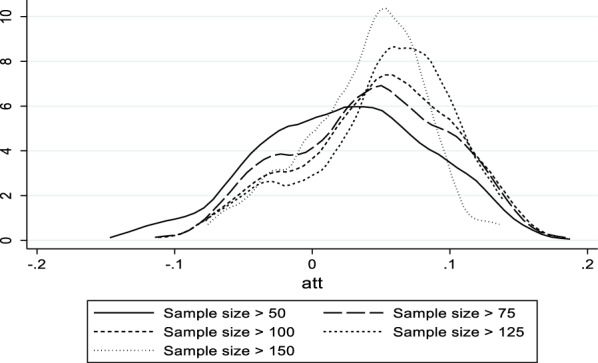
Density functions of ATTs across profiles by minimum sample size

Figure 4 shows the distribution of ATT across subsamples with > 50, > 75, > 100, > 125 and > 150 treatment observations. The figure clearly shows that the ATT distributions are not centered around zero, which would be expected if child diet diversity has no systematic relationship with stunting. The distributions are skewed to the right in the sense that positive estimated ATTs are more likely observed than negative for any minimum sample size, corroborating theory and previous empirical evidence. Finally, the distribution of ATT shifts to the right with increasing sample size and the percentage of estimated negative ATTs becomes low.^28^ Therefore the figure passes each of the tests on whether the estimated nonzero treatment effects are not simply due to sampling variation and We conclude that there is clear evidence that inadequate child diet diversity increases the probability of stunting, but not for all children. This finding also corroborates the premise that the agriculture-food-nutrition nexus is complex and that the effect of diet diversity is context-dependent.

The interesting question is now which profiles show the largest impact coverage (lowest exclusion error) and/or lowest inclusion error. However, due to the presence of sampling variation, we face a trade-off in terms of the minimum profile sample size and the false discovery rate (FDR) as limiting the analysis to profiles with larger sample sizes reduces the false discovery rate but also the available number of profiles. In Additional file 5 we show that focusing on profiles with at least 100 observations and an ATT with t-value of 1.645 or higher nearly minimizes the FDR while keeping a relatively high number of profiles (36).

Table 4 summarizes the characteristics of these profiles. The table shows that the vast majority of the significant profiles include children of 6–12 months old, which is consistent with the literature emphasizing the first “1000 days”, from conception to the second birthday of the child, where breastfeeding is important in the first 4–6 months, after which a shift towards a healthy diet should take place to avoid long term irreversible health damage (e.g. [3]. The evidence suggests that a shift to a healthy diet is already important right after breastfeeding. Improving diet diversity is also key in rural areas where impacts are higher possibly because diet diversity is generally higher among the urban children and other constraints have become binding. Larger impacts could also reflect that urban diets arguably are more diverse even if they are inadequate according to the household diet diversity scores. The agro-ecological variables (slope, length of growing period, farming system) point at areas where food production is facing challenges: semi-arid conditions and hilly landscapes, sometimes with only a small part of the land available for production, and a heavy reliance on maize production, which is likely to translate into an equally heavy reliance on maize for consumption, particularly because households are very likely to be poor with little alternative income as mothers are not working and multiple small children are present. Of course, given the complexity of the agriculture-food-nutrition nexus it is difficult to infer the exact mechanism underlying the variations in impact without further research, but this does not mean that we (and decision-makers) can ignore the large heterogeneity in impacts.

**Table 4:** Characteristics represented in profiles with significant estimated ATT effects

	Variable	Value	% of profiles
1	Location	Rural	75
2	Age of child	6–12 Months	72
3	Farming system	Maize/mixed farming system	72
4	Length growing period	121–180 Days	67
5	Working status mother	Not working	58
6	Slope	8–30 Degrees	53
7	Has sibling(s) ≤ 5 years	Yes	50
8	Wealth tercile	Poorest	36
9	Education	Secondary	17

All Characteristics that occur in the profiles with minimum sample size of 100, a positive estimated ATT and t-value of 1.645 or higher are reported

Profiles of targeting groups vary in terms of impact coverage (exclusion error) as well as inclusion error and a policy-maker may want to focus on the profile that has the largest impact coverage and the lowest inclusion error. The “winning” impact profiles that minimize the weighted geometric average for different values of $\alpha$ are presented in Additional file 6, showing that there is a partial but certainly not perfect overlap in the winning polling and impact profiles (cf. Additional file 6: Tables S.6.1 and S.6.2).

### Are the neediest also benefitting the most from promoting diet diversity?

**Fig. 5:**
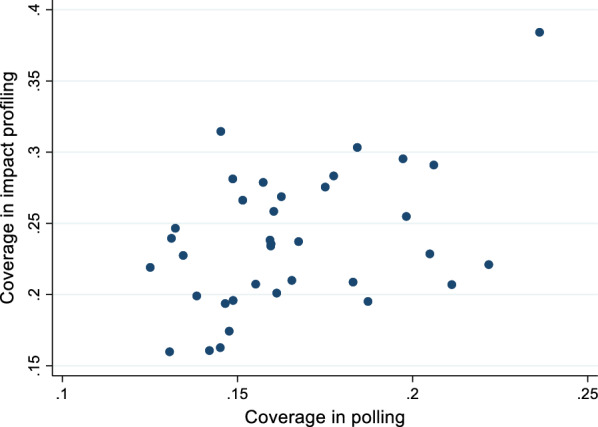
Scatter plot of coverage in polling versus impact profiling approach

We are now finally in the position to answer our last question, namely to which extent a polling approach focusing on the neediest will be equivalent to focusing on impact. For each of the profiles with a significantly estimated positive ATT we can calculate both the coverage (see Box 1) as well as the impact coverage (Eqs. 2 and 3). If these different measures are strongly correlated, then the polling and impact profiling approaches yield similar results.^29^ Figure 5 shows a scatter plot of the coverages across impact profiles with a significantly estimated positive ATT effect (t-value exceeding 1.645) and minimum sample size of 100. The correlation coefficient is 0.44.^30^ This suggests that targeting profiles with a high share of households with children suffering from inadequate diet diversity (profiles with high coverage) will not be efficient as profiles with high coverage do not always have a high impact coverage. The divergence between polling and impact profiles suggests that there are other binding constraints on reducing stunting that need to be overcome apart from inadequate diet diversity, and targeting on need only will be wasteful.

## Discussion

A common policy challenge is to appropriately target policy interventions when impacts are heterogeneous due to complex interaction effects. There is an increasing emphasis on evidence-based policy-making but the available evidence is typically not informative on how interventions ‘ impacts differ across treatment units. For this reason, policy-makers may rely on the ‘profiling’ approach to identify who is in ‘need’, effectively assuming that impacts and need are strongly correlated.^31^

In this paper we introduce the novel method of impact profiling to identify groups of treatment units that stand to benefit most from a given policy intervention, i.e. an extension of the profiling approach to identify who has the most ‘impact’. This methodology was used to analyze which households with children between 6–25 months will benefit most from an improvement in diet diversity in terms of a reduction in child stunting in Zimbabwe. This methodology has wide applicability beyond the chosen study locale/country (c.q. Zimbabwe) and indicators (c.q. diet diversity and stunting), however. It can equally be applied to identify (impact) profiles for other health and nutrition indicators and relationships (e.g. wasting, children underweight, malaria, diarrhea, anemia, Vitamin A deficiency, etc.) and socio-economic indicators and relationships (e.g. unemployment, poverty, gender indicators).

Our application of the (impact) profiling method implies that it is important to allow for the complexity of empirical relationships that are commonly estimated, that this is feasible, and that this enhances the policy-relevance of estimated empirical relationships. We found that the diet diversity-stunting relationship is complex, and that the impact varies across profiles (groups of households with children). We also established that the profiling approach focusing on need is positively correlated with but often diverges from the impact profiling approach focusing on impact—the profiles with a high coverage of households with children suffering from inadequate diet diversity do not always have a high impact coverage. Therefore policy-makers need to remain alert that targeting on need is not always the same as targeting on impact, and any divergence suggests that there are other binding constraints on child growth besides inadequate diet diversity.

## Conclusions

Estimation of heterogeneous treatment effects does not only benefit policy-makers by increasing the efficiency of targeting. It is also important by increasing the external validity of the estimated impact findings [12]. We found that the impact of child diet diversity on stunting depends on various agro-ecological variables, which allows policy-makers to upscale our findings to sites outside our study area (c.q. Zimbabwe) but with similar agro-ecological conditions. Of course, further research is needed to confirm the external validity of the findings, but generalizing findings across relatively homogenous areas in terms of the relevant conditioning variables is plausibly more appropriate than across highly heterogeneous areas.

Here we have shown that targeting on need is not always efficient, as the impact of diet diversity on stunting is often larger for less needy households. This discrepancy can reflect that increases in diet diversity may have relatively fewer nutritional benefits among many households with initially low (and inadequate) levels of diversity because their diet remains mostly centered around maize and beans. Also, many households with children suffering from inadequate diet diversity may be facing other binding constraints that need to be overcome, such as the prevalence of diseases and parasites which lower the impact of diet improvements.

It is interesting to note that our method can be further developed to incorporate equity concerns by using a weighted average or even a social welfare/inequality function of the individual households’ outcomes following an intervention. In that case, targeting on impact may create an efficiency-equity trade-off such that targeting on need may actually be more equitable than targeting on impact. Of course, whether this is the case will depend on the circumstances at hand and how equity is defined.

Also in this paper we applied the (impact) profiling approach to a single cross-sectional dataset [6]. It would also be possible to extend the analysis to multiple cross-sections to study the presence of structural breaks in the estimated relationships. For instance, one could identify the success of an intervention targeting the needy by the extent to which winning profiles in the polling approach are altered subsequently or how winning profiles change over time with changes in socio-economic contexts (e.g. economic crises).

Finally, it is important to note that this paper has not addressed one important issue, namely *which* policies are most appropriate to increase child diet diversity. In this paper we have shown that a pro-poor policy of increasing child diet diversity in rural areas with a medium length of growing period, focusing on non-working mothers in the poorest tercile with multiple or very young children, will have a relatively large impact in Zimbabwe. But the paper has been silent on which policy instruments will be most appropriate, such as through the introduction of new crops, awareness training, subsidies, etc. Targeting interventions at winning impact profiles is recommended as the impact of improvements in child diet diversity on stunting will be relatively large, but the choice of intervention remains an important issue to consider. The optimal choice of intervention(s) will not only depend on the expected impacts, but also on their relative costs as well as the relative cost of targeting different profiles. Finally, we have to acknowledge the fact that policy makers do not always base their policies on analyses like the one presented in this paper; in Zimbabwe, it is clear that targeting has been politically motivated to a large extent. However, even under such conditions, analyses on potential impact can clarify the costs of such politicization to society.

## Supplementary information

**Additional file 1:** Correcting for nonrandom interview timing.

**Additional file 2:** Spatial attributes.

**Additional file 3:** Selected variables and univariate (parametric) analysis.

**Additional file 4:** Additional results for matching.

**Additional file 5:** Signs of estimated ATT effects and trade-off minimum profile sample size versus FDR.

**Additional file 6:** Winning profiles in (impact) profiling.

## Data Availability

The spatially explicit DHS data that support the findings of this study are available from https://www.dhsprogram.com/ but must be requested from the DHS. Spatial attributes are available online from IASA/FAO (https://www.gaez.iiasa.ac.at/).

## Abbreviations

ATE: Average Treatment Effect
ATT: Average Treatment Effect on the Treated
ATU: Average Treatment Effect on the Untreated
CDDS: Child Dietary Diversity Score
DHS: Demographic and Health Survey
FDR: False Discovery Rate

## Footnotes

1 Measured by the proportion of children under 5 years who were moderately (severely) underweight, i.e. have a weight for age below −2 (−3) standard deviations of the average.

2 https://www.worldbank.org/en/country/zimbabwe

3 https://www.hrw.org/news/2019/03/29/zimbabwe-should-investigate-allegations-aid-abuse

4 This inverted U-shape pattern of stunting relative to age is a common finding for many developing countries. E.g. [4] note that “In many such settings, prevalence starts to rise at the age of about three months, the process of stunting slows down at around three years of age, after which mean heights run parallel to the reference. Therefore, the age of the child modifies the interpretation of the findings: for children in the age group below 2–3 years, low height-for-age probably reflects a continuing process of ‘failing to grow’ or ‘stunting’; for older children, it reflects a state of ‘having failed to grow’ or ‘being stunted’” (p. 46).

5 We note that there does not seem to be a clear spatial correlation with the political “landscape” (Zanu PF’s traditional strongholds Midlands, Mashonaland West, East and Manicaland and opposition strongholds Bulawayo, Matabeleland North and South show a varied stunting pattern), suggesting that the above-mentioned politicization of resource allocation is overridden by other, locational factors.

6 Here one may add a third issue, namely how can we increase diet diversity? In this paper we will assume that ‘interventions’ exist which can increase diet diversity without analyzing the appropriate choice of policy instruments to implement these in practice. We revert to this point in the conclusions of the paper.

7 We also note that the DHS does not include direct information on caloric intake.

8 An alternative approach is to apply machine learning algorithms.

9 Note that we assume that *x* and *y* take on a finite number of integer values or that real values can be classified to yield a finite number of class values. The independent variable *y* can also be a vector where each element can have multiple (integer) values.

10 A weighted binary response model will also not take into account inclusion and exclusion errors as it only models the probability of an outcome (c.q. inadequate diet diversity) conditional on the value of *x*.

11 E.g. a score of 2 could be the results of consuming grains and pulses, or of consuming grains and vegetables/fruits. Obviously, the latter diet would be more diverse than the former, especially if the consumed items are maize and beans only.

12 This set of independent variables can be the same as those selected for the polling approach but this is not necessarily the case.

13 Alternatively, if one prefers to define treatment as a change from inadequate to adequate child diet diversity, then ATT should be replaced by ATU in the methodology. The empirical results will be the same (though of opposite sign).

14 Impact is measured here as the sum total of individual household impacts. In principle, it would also be possible to include distributive considerations by using a weighted average or even a social welfare/inequality function of individual households’ outcomes following the intervention.

15 Another reason for targeting will be the relative costs of reaching different profiles. This is not taken into account in the current analysis.

16 Ideally, of course, we would have information about the child’s history of diet diversity as stunting will reflect its cumulative effect. Because this information is unavailable, we use the 24 h recall as a proxy for the diet diversity experienced by the child so far. Given that this proxy will be noisy, the observed link with stunting is most likely underestimated (attenuation bias).

17 First, attribute maps were rasterized at 30 arc seconds resolution (app. 1 km) and resampled to a similar georeference (zwe_adm2). Second, maps were exported in vector form (latitude, longitude and attribute) to SAS software and merged with coordinates and identifiers of DHS data points. Third, for each DHS data point the center of the nearest neighbor grid cell was identified which related to all spatial attributes. Additional file 2 includes detailed descriptions of the individual spatial attributes.

18 We verified that the excluded 8 variables were also jointly insignificant (joint F-test, p-value 0.16).

19 It is true that the use of multiple waves would allow for the study of structural breaks in the estimated relationships but this falls outside the scope of this paper (see conclusions).

20 To be precise, in that paper, slightly different concepts are defined and used to define the winning profile: (1) edge: the ratio of the probability of winning combination and the probability of second best, (2) coverage (see Box 1), and the (3) uniqueness of combination of x-variable outcomes for y value.

21 Or ‘largest association with improved diet diversity for small children’ in case it is difficult to make causal inferences (see our caveat before).

22 As noted before, to the extent that diet diversity (and stunting) is affected by other (unobserved) variables, one should be cautious about making causal inferences of course.

23 We are grateful to one of the referees for pointing this out.

24 The semi-reduced form approach implies that we study the impact of a change in diet diversity on stunting allowing for simultaneous changes in calorie intake *in line with existing dietary patterns in Zimbabwe*, where the latter are conditional on the included control variables and hence allowing for variation across different types of children, households and locations (e.g. by child age, household wealth status, farming system). Because we are unable to control for calorie intake itself, the empirical analysis does not address the question how stunting would change with changes in diet diversity if diets deviating from the existing diversity-calorie nexus are introduced.

25 See Sect. 2.

26 Table 3 reports approximate standard errors. Bootstrapped standard errors give similar results (s.e. of ATT becomes 0.02 instead of 0.03). The ATT reported is unweighted for sampling weights, but weighting does also not affect the results (ATT remains 0.01).

27 Another interpretation is that the result is due to sampling error. The estimate is based on 859 matched treatment observations, however.

28 Additional file 5: Figure S.5.1 shows the number of profiles with positive and negative estimated ATTs and an (absolute) t-value of 1.645 or higher. This figure clearly shows that the number of negative estimated ATTs quickly falls with increasing minimum sample sizes, while the decline of the number of positive estimated ATTs is much less pronounced. The total number of estimated ATTs falls with increasing sample sizes as smaller-sized profiles are excluded. The increasingly smaller number of profiles available plausibly also explains why the rightward distributional shift is no longer observed for sample sizes in excess of 150 (only 22 profiles remain from the 671 initially available with sample size in excess of 50).

29 We ignore differences in inclusion error here because we do not have a separate measure for the inclusion error in the impact profiling approach (see Sect. 3). This would require the identification of actual policy experiments and how they affect diet diversity, both of children with inadequate as well as adequate diet diversity, where the latter will create an ‘impact’ inclusion error. Also we limit the comparison of the polling and impact approach to profiles with a minimum sample size of 100 for which significant positive ATT effects have been estimated as we do not have reliable (expected to be positive) ATT estimates for other profiles because of too small sample sizes.

30 The Spearman rank correlation is 0.37.

31 Although Wesenbeeck and Sarpong (forthcoming) concluded in their review paper on SSA policies aimed at improving diet diversity and food security that many policies focus on enhancing production of food, with assumed impact pathways leading to improved diet diversity. The record on actual impacts has been mixed. On the other side of the spectrum, there is a global focus on mother and child health (the first 1000 days approach), but evidence on targeted interventions in SSA countries is lacking. Hence, in both approaches, policies would benefit from more empirical evidence on the impact of targeted interventions.
