# Supplementary material for: Who lacks and who benefits from diet diversity: evidence from (impact) profiling for children in Zimbabwe
Source: Int J Health Geogr. 2020 Nov 4;19:45. doi: 10.1186/s12942-020-00240-2 (PMC7640455; doi:10.1186/s12942-020-00240-2)
Supplement: Supplementary file 1 — Additional file 1: Correcting for nonrandom interview timing. [file 12942_2020_240_MOESM1_ESM.pdf]

Additional File 1: Correcting for nonrandom interview timing

The DHS survey is a cross-sectional data set measuring diet diversity in the past 24 hours at the time of the household interview. Because the survey was implemented during the 5-month period of July-December, an artificial temporal pattern in measured diet diversity will arise if time of interview is nonrandom and correlated with other variables that are also related to diet diversity ('confounders'). Upon inspection, we found a clear temporal pattern in the collection of the data (see Figure S.1.1), while also a time-dependent rural/urban coverage was found.

Figure S.1.1 Temporal dispersion of clusters in space, colors indicate month of visit

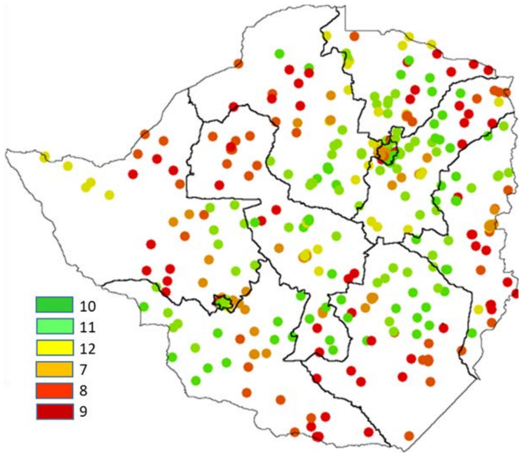

Hence, we need to correct for these temporal patterns in measured diet diversity to avoid distorting the conclusions on diet diversity. We therefore first regressed measured diet diversity at the time of the interview on a wide range of possible determinants *as well as* the day of interview (including its square). As determinants we chose the top-10 variables that turned out to be most powerful in univariate regressions (see below for details). The (squared) day of the interview was included to capture any independent seasonal pattern in diet diversity, e.g. as a result of agricultural seasons. Next we imputed the diet diversity for each household by predicting its value if the household would

have been interviewed at the start of the survey period (i.e. on day 1). The regression results are presented in the table below.

Table S.1.1. OLS estimates of measured diet diversity score controlling for time of interview

	Estimate	s.e.
Age of child (years)	**2.99*****	-0.44
Age of child squared	**-0.71*****	-0.18
Child has sibling(s) of ≤5 years (dummy)	**-0.12***	-0.06
Mother is working in the last 7 days (dummy)	**0.24*****	-0.07
Education of mother (omitted category: 'No education')		
Primary (dummy)	0.15	-0.32
Secondary (dummy)	0.29	-0.32
Tertiary (dummy)	0.51	-0.35
Wealth quintile (omitted category: 'Bottom quintile')		
2nd quintile (dummy)	0.08	-0.09
3rd quintile (dummy)	0.12	-0.09
4th quintile (dummy)	**0.40*****	-0.1
5th quintile (dummy)	**0.33*****	-0.11
Urban (dummy)	**0.19****	-0.09
Land use (omitted category: '>50% cultivated land')		
>50% forest/barren land (dummy)	**0.60*****	-0.19
>50% grass and wood land (dummy)	**0.54*****	-0.18
>50% built up land (dummy)	**0.67*****	-0.2
land cover associations (dummy)	**0.69*****	-0.2
Length of growing period (omitted category: '0-75 days')		
76-120 days (dummy)	-0.07	-0.18
121-180 days (dummy)	-0.05	-0.19
>180 days (dummy)	-0.25	-0.28
Farming systems (omitted category: 'Highland temperate mixed')		
Root crop/Cereal-root crop mixed (dummy)	-0.49	-0.35
Maize mixed (dummy)	0.09	-0.26
Large commercial and smallholder/Pastor (dummy)	-0.41	-0.43
Agropastoral millet sorghum (dummy)	-0.07	-0.3
Slope 8-30 degrees (dummy)	0.04	-0.11
Slope > 30 degrees (dummy)	0.12	-0.18
Day of interview (1-167)	**-0.01****	0.00
Day of interview squared (10^{-4})	**0.38****	0.18
N	1741	

Notes: Inference: *** p<0.01; ** p<0.05; * p<0.10. Coefficients significant at 10% are in bold.

Using the OLS estimates, for (only) 3 households the predicted value was below zero or above 7, and in these cases the imputed value was set equal to zero respectively 7.
