# Supplementary material for: Who lacks and who benefits from diet diversity: evidence from (impact) profiling for children in Zimbabwe
Source: Int J Health Geogr. 2020 Nov 4;19:45. doi: 10.1186/s12942-020-00240-2 (PMC7640455; doi:10.1186/s12942-020-00240-2)
Supplement: Supplementary file 2 — Additional file 2: Spatial attributes. [file 12942_2020_240_MOESM2_ESM.pdf]

Additional File 2: Spatial attributes

Below we briefly describe the spatial attributes for Zimbabwe that were used in this study. Table 1 gives an overview.

Table A.2.1. Spatial attributes, units and sources

Attribute	Unit	Source
Length of Growing Period	Days	IIASA/FAO, 2012
Soil Suitability High Input Level	Classes	Ibid
Soil Suitability Low Input Level	Classes	Ibid
Slope	Classes (%)	Ibid
Altitude	M	Ibid
Dominant Land Use	Categories	Ibid
Farming Systems	Categories	FAO/ World Bank (2001)
Administrative map	Polygons	GADM, accessed 2018

Length of Growing Period

The Length of Growing Period (LGP) is an important indicator for agricultural development as it reflects the number of consecutive days when temperature and soil moisture are conducive to crop growth and development. The LGP is based on water balance calculations for a reference crop using evapotranspiration, precipitation and soil specific information to assess a continuous period that moisture is available in the soil matrix. About 42 per cent of the surface area falls within the LGP of 120-180 days range that enables cultivation opportunities for a wide range of rainfed crops. The LGP class of 90-120 days, occupying 11 per cent of the area, covers an excellent time period to cultivate drought resistance crops like millet and sorghum. Spatial distribution of LGP's is presented in Figure A.2.1.

Soil suitability (low and high inputs)

Soil suitability reflects the degree of constraining soil characteristics for crop and fodder cultivation under different input levels. Main soil qualities considered for soil suitability assessment are: nutrient availability, nutrient retention, rooting conditions, oxygen, excess salts, toxicity and

workability. Using a set of functional relationships that accommodate individual soil characteristics, a soil suitability is calculated for low and high input farming. Zimbabwe has excellent soils for high input level farming with a total of 32 per cent in the three highest suitability categories. Suitability under low input levels has it gravity point towards medium and moderate suitability classes (Figures A.2.2, A.2.3)

Slope/Altitude

Elevation and slopes for Zimbabwe were derived from a 3 arc second (app. 90 meters) DEM compiled by the NASA Shuttle Radar Topographic Mission (SRTM). Data were processed to derive 30 arc-seconds information on prevailing altitudes (Figure A.2.4) and slope classes (Figure A.2.5). 4 percent of the area has slopes below 2 per cent which makes these sites suitable for crop cultivation without additional measures, 15 percent falls in the 2-8 percent slopes which requires substantial soil conservation schemes to make these areas arable. Slope from 8-16 are only suitable for permanent crops and forestry. Higher slopes should be excluded from cultivation and fall under a regime of protected areas.

Farming systems

Farming systems characterize groups of individual farms that apply the same set of interventions to control a broadly similar natural resource base. From the policy point of view, these groups can tune and upscale comparable development strategies. Rice-Tree crop (15 per cent) and Highland crops (29 per cent) are the largest farming systems in Zimbabwe (Figure A.2.6).

Dominant land use

The seven land use/land cover categories that were used characterize each 5 arc-minute grid-cell, are: Rain-fed cultivated land, Irrigated cultivated land, Forest, Grassland and other vegetated land,

Barren and very sparsely vegetated land, Water and Urban land and land used for housing and infrastructure. (Figure A.2.7).

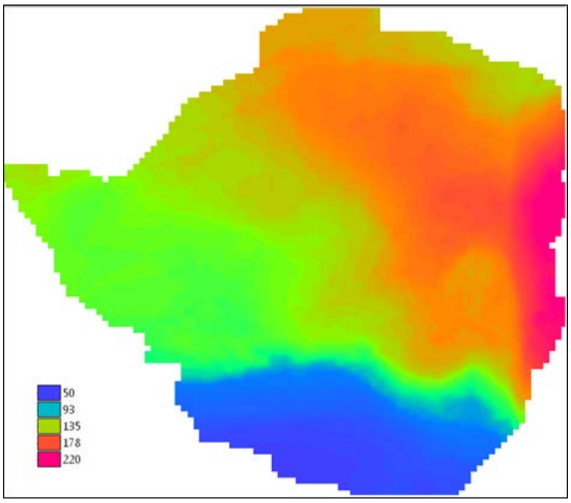

Figure A.2.1 Length of Growing Period in days

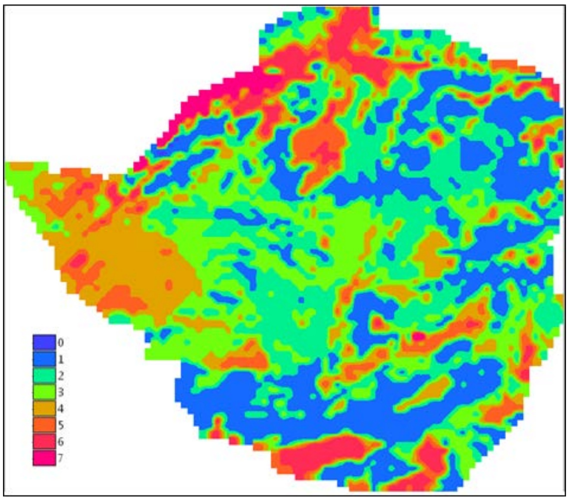

Figure A.2.3 Soil suitability at high input level. 1=>75%: very high, 2=>63%: high, 3=>50%: good, 4=>35%: medium, **very high, 2=>63%: high, 3=>50%: good, 4=>35%: medium,** **5=>20%: moderate,6=>10%: marginal. 7=>0%: very marginal, 8=Unsuitable**

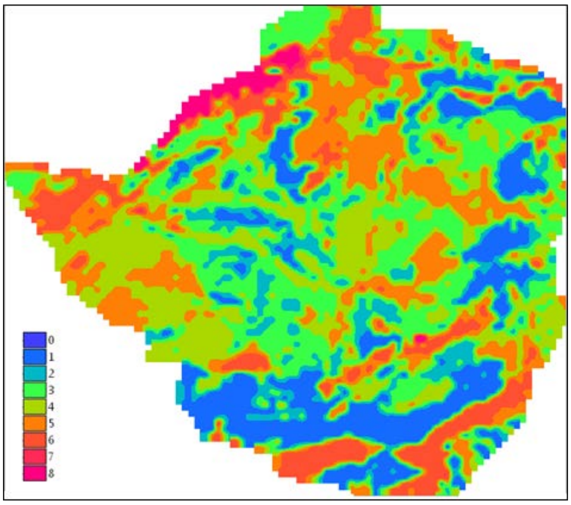

Figure A.2.2 Soil suitability at low input level. 1=>75%: 5=>20%: moderate,6=>10%: marginal. 7=>0%: very marginal, 8=Unsuitable

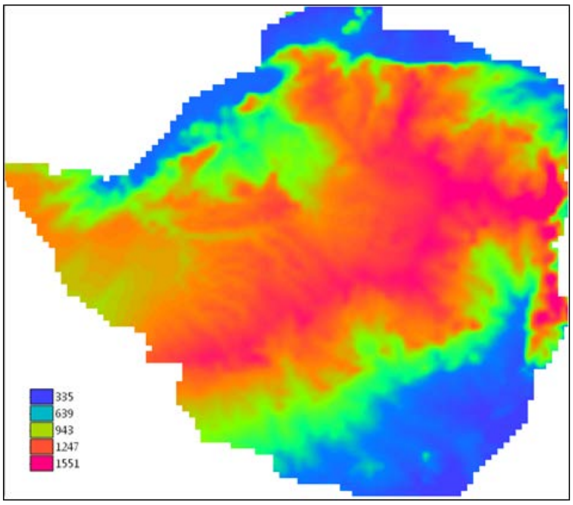

Figure A.2.4 Altitude in meters

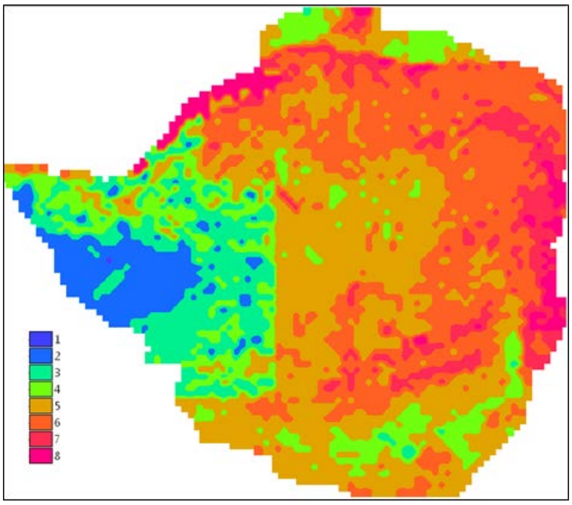

Figure A.2.5 Slope classes

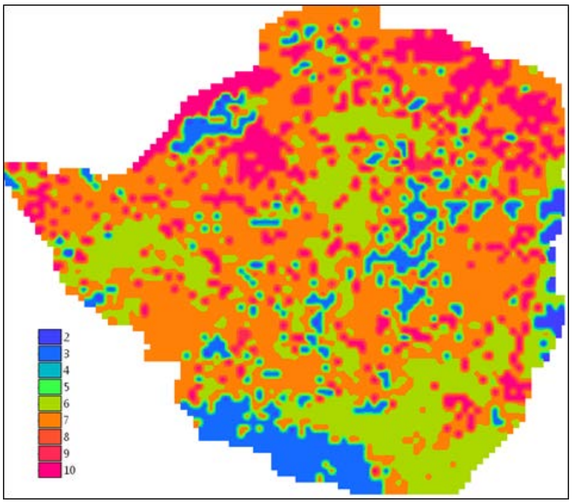

Figure A.2.7 Dominant land use. 2= > 75% forest, 3= > 75% grass and woodland, 4= >75% barren land, 5= 50-75% cultivated land, 6= 50-75% forest, 7= 50-75% grass and woodland, 8= 50-75% barren land, 9= >50% built-up land, 10= land cover associations.

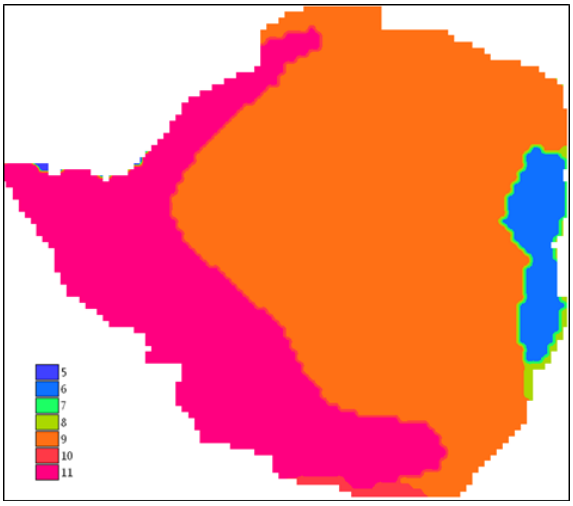

Figure A.2.6 Farming systems. 5 = Highland perennial, 6 = Highland temperate mixed,7 = Root crop, 8 = Cereal-root crop mixed, 9 = Maize mixed, 10= Large commercial & smallholder, 11= Agro-pastoral millet/sorghum
