# Supplementary material for: Who lacks and who benefits from diet diversity: evidence from (impact) profiling for children in Zimbabwe
Source: Int J Health Geogr. 2020 Nov 4;19:45. doi: 10.1186/s12942-020-00240-2 (PMC7640455; doi:10.1186/s12942-020-00240-2)
Supplement: Supplementary file 3 — Additional file 3: Selected variables and univariate (parametric) analysis. [file 12942_2020_240_MOESM3_ESM.pdf]

Additional File 3: Selected variables and univariate (parametric) analysis

The set of variables included as potentially relevant for diet diversity including the potential outcomes for these variables is summarized in Table S.3.1. Outcomes are grouped to create the classes used in the analyses and hence do not necessarily follow classes defined in the DHS or other databases used.

Table S.3.1. Longlist of variables for the analysis

	**Variable name**	**Outcomes**
1	Age of child	6-12 months, >12 months
2	Gender of child	Male, female
3	Child has sibling(s) of ≤5 years	No, yes
4	Age of mother	$< 35, \geq 35$
5	Education of mother	No education, primary, secondary, higher
6	Marital status	Never in union, married, living with partner, widowed, divorced, no longer living together/separated
7	Mother is currently working	No, yes
8	Age of household head	$< 45, \geq 45$
9	Sex of household head	Male, female
10	Wealth quintile (urban/rural specific)	Poorest/poorer, middle, richer/richest
11	Location	Urban, rural
12	Farming system	Irrigated, tree crop, forest based, rice-tree crop, highland perennial, highland temperate mixed, root crop/cereal-root crop mixed, maize mixed, large commercial and smallholder/pastoral, agropastoral millet sorghum, sparse (arid), coastal artisan fisheries, irrigated area in rainfed farming system
13	Land use	>50% Cultivated land, > 50% forest/barren land, >50% grass and woodland, >50% built up land, land cover associations
14	Suitability under high input use	>75% Very high, >63% high, >50% good, >35% medium, >20% moderate, >10% marginal, >0% very marginal, unsuitable, water
15	Suitability under low input use	>75% Very high, >63% high, >50% good, >35% medium, >20% moderate, >10% marginal, >0% very marginal, unsuitable, water
16	Length of Growing Period (LGP)	0-75 Days, 76-120 days, 121-180 days, >180 days
17	Altitude	0-500 m, 500-1000 m, 1000-2000 m, > 2000 m
18	Slope	0-8%, 8-30%, >30%

Table S.3.2. Summary statistics of variables for analysis

Variable	Obs	Mean	Std.	Dev.	Min
Stunted (dummy)	1,613	0.23	0.42	0	1
Inadequate diet diversity (dummy)	1,613	0.54	0.50	0	1
Age of child (years)	1,613	1.24	0.43	0.50	2.08
Gender of child (dummy, male=1)	1,613	0.51	0.49	0	1
Child has sibling(s) of ≤5 years (dummy)	1,613	0.55	0.50	0	1
Age of mother (dummy, ≥ 35 years=1)	1,613	0.14	0.34	0	1
Education mother (omitted: `none’)					
Primary (dummy)	1,613	0.33	0.47	0	1
Secondary (dummy)	1,613	0.61	0.49	0	1
Tertiary (dummy)	1,613	0.04	0.19	0	1
Marital status (omitted: ‘Never in union’)					
Married (dummy)	1,613	0.84	0.37	0	1
Living with partner (dummy)	1,613	0.04	0.19	0	1
Widowed (dummy)	1,613	0.01	0.11	0	1
Divorced (dummy)	1,613	0.03	0.17	0	1
No longer living together/separated (dummy)	1,613	0.04	0.19	0	1
Mother is currently working (dummy)	1,613	0.38	0.49	0	1
Age of household head (dummy, ≥ 45 years=1)	1,613	0.24	0.43	0	1
Sex of household head (dummy, male=1)	1,613	0.63	0.48	0	1
Wealth quintile (omitted: ‘Poorest’)					
2nd Quintile (dummy)	1,613	0.21	0.40	0	1
3rd Quintile (dummy)	1,613	0.20	0.40	0	1
4th Quintile (dummy)	1,613	0.15	0.36	0	1
5th Quintile (dummy)	1,613	0.16	0.36	0	1
Urban (dummy)	1,613	0.26	0.44	0	1
Farming system (omitted:`Highland temperate mixed’)					
Root crop/Cereal-root crop mixed	1,613	0.01	0.11	0	1
Maize mixed	1,613	0.79	0.41	0	1
Large commercial and smallholder/Pastor	1,613	0.00	0.06	0	1
Agropastoral millet sorghum	1,613	0.11	0.31	0	1
Land use (omitted: `>50% Cultivated land’)					
> 50% forest/barren land	1,613	0.32	0.47	0	1
>50% grass and woodland	1,613	0.38	0.49	0	1
>50% built up land	1,613	0.14	0.35	0	1
Land cover associations	1,613	0.13	0.33	0	1
Suitability under high input use (omitted: `>75% Very high’)					
>63% high	1,613	0.37	0.48	0	1
>50% good	1,613	0.18	0.39	0	1
>35% medium	1,613	0.07	0.25	0	1
>20% moderate	1,613	0.03	0.17	0	1
>10% marginal	1,613	0.04	0.19	0	1
Water	1,613	0.00	0.00	0	0

Suitability under low input use (omitted: ` >50% good/high/very good')					
>35% medium	1,613	0.25	0.43	0	1
>0% (very) marginal/moderate	1,613	0.24	0.43	0	1
Length of Growing Period (omitted: `0-75 Days')					
76-120 days	1,613	0.13	0.34	0	1
121-180 days	1,613	0.71	0.45	0	1
>180 days	1,613	0.11	0.32	0	1
Altitude (omitted: `0-500 m')					
500-1000 m (dummy)	1,613	0.27	0.44	0	1
1000-2000 m (dummy)	1,613	0.66	0.47	0	1
Slope (omitted: `0-8%')					
8-30%	1,613	0.74	0.44	0	1
>30%	1,613	0.12	0.33	0	1

Note: figures are weighted with sampling weights

Ranking of variables by p-value is presented in Table S.3.3, where the 10 selected variables are indicated with an "*" in the final column (with p-value<0.10).

Table S.3.3. Top-10 of variables selected

	Variable name	p-value	R squared	Selected
1	Age of child	0.000000	0.101	*
2	Working status of mother	0.000000	0.021	*
3	Education	0.000001	0.018	*
4	Wealth quintile (urban/rural specific)	0.00001	0.013	*
5	Child has sibling(s) of ≤5 years	0.00002	0.011	*
6	Location	0.00002	0.011	*
7	Land use	0.001	0.010	*
8	Length of Growing Period (LGP)	0.02	0.006	*
9	Farming system	0.04	0.006	*
10	Slope	0.09	0.003	*
11	Suitability under high input use	0.16	0.005	
12	Altitude	0.30	0.001	
13	Marital status	0.31	0.003	
14	Suitability under low input use	0.34	0.001	
15	Age of mother	0.57	0.000	
16	Sex of household head	0.58	0.000	
17	Gender of child	0.59	0.000	
18	Age of household head	0.95	0.000	
