# Supplementary material for: Who lacks and who benefits from diet diversity: evidence from (impact) profiling for children in Zimbabwe
Source: Int J Health Geogr. 2020 Nov 4;19:45. doi: 10.1186/s12942-020-00240-2 (PMC7640455; doi:10.1186/s12942-020-00240-2)
Supplement: Supplementary file 4 — Additional file 4: Additional results for matching. [file 12942_2020_240_MOESM4_ESM.pdf]

Additional File 4: Additional results for matching

Figure S.4.1. Common support across treated and untreated observations

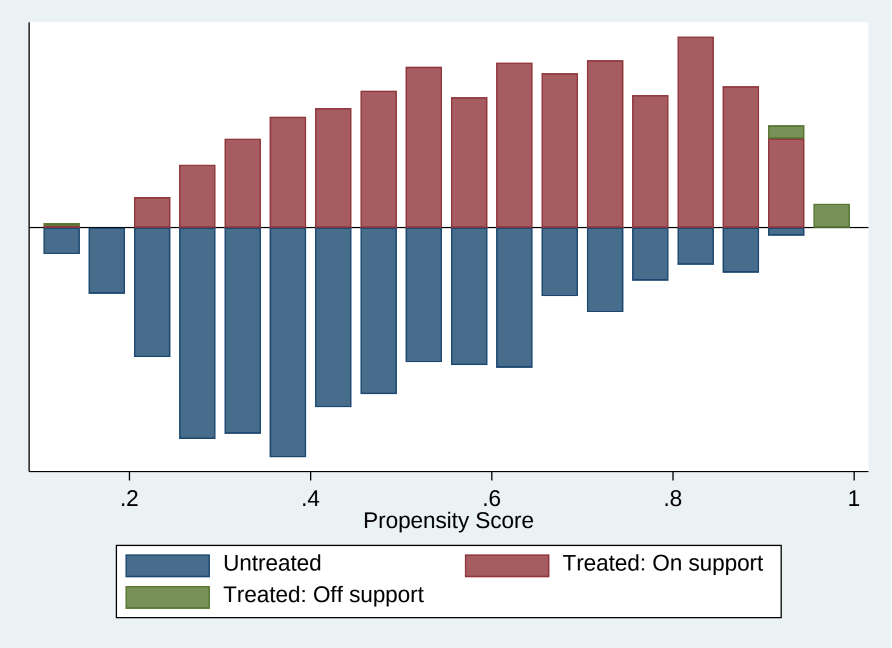

The propensity score method assumes that there is 'balancing' in the sense that the propensity score should have a similar distribution ("balance") in the treated and comparison groups. For this, it is common practice to split the sample by propensity score quintiles first and to check whether the differences in proportions of treatment and comparison groups are not statistically significant. If this is not the case, then one or more quintiles can be split in smaller blocks to see whether balance can be achieved. If not, then the propensity score model needs to be re-specified (Imbens 2004). In our case, balance was achieved by splitting the 4th quintile into two blocks, creating 6 blocks (Table S.4.1).

Apart from balancing of propensity scores across treatment and comparison groups, balancing is also required for covariates across treatment and comparison groups. Although balance in observed covariates does not necessarily indicate balance in unobserved covariates, balance in the former plausibly gives also some support for the claim that unmeasured covariates will not be confounders biasing the treatment effect estimates. While it is possible to test for balancing before applying a matching procedure, any lack of balance is not necessarily problematic as matching will eliminate out most of these differences.

Table S.4.1 Propensity score blocks required for balancing scores across treatment and comparison groups

Propensity score (range)	Number of observations		
	Treated	Comparison	Total
[0.0-0.2)	38	2	40
[0.2-0.4)	294	135	429
[0.4-0.6)	234	252	486
[0.6-0.7)	79	147	226
[0.7-0.8)	52	138	190
[0.8-1.0]	34	211	245
Total	731	885	1,616

Table S.4.2 presents the results for different types of balancing tests, namely the standardized percentage bias, t-tests for equality of means in the two samples, and the variance ratio, both for the unmatched and matched samples. Imbalance in some covariates is expected, of course (even in RCTs), as exact balance is a large-sample property. Looking first at the t-tests, none of the covariates in the matched samples are significant at 10%. When we consider the two other types of balancing tests, there is also little reason for concern. The reported standardized percentage biases before and after matching (Rosenbaum and Rubin, 1985) show large reductions in the (absolute) bias achieved. Although there are no rules about what is acceptable bias, proposed maximum standardized differences range from 10 to 25% (Austin 2009, Stuart *et al.* 2013). For the matched samples we find standardized percentage biases of just 4.6% or less.

The ratios of variances in the propensity scores between the treated and comparison groups are close to one for the matched samples (for the linear and square terms of age of the child)[1]. This ratio should equal 1 if there is perfect balance. Our ratios therefore fall comfortably within the 95% confidence interval (see Austin, 2009). Furthermore, the pseudo R^2 from probit estimation of the propensity score on all the variables is close to zero for the matched sample (0.004), as expected with proper matching. The LR test also rejects joint significance of all the regressors (p-value 0.997).

[1] These measures are only available for continuous variables.

Table S.4.2 Balancing tests for covariates (after matching)

	Unmatched Matched	Treated	Control	%bias	% less |bias|	t	p>|t|	V(T)/V(C)
Age of child	U	1.11	1.39	-69.3		-13.8	0.00	1.20*
	M	1.11	1.13	-2.90	95.8	-0.60	0.55	1.05
Age of child squared	U	1.40	2.08	-65.6		-13.2	0.00	0.94
	M	1.42	1.44	-1.80	97.2	-0.39	0.70	1.01
Child has sibling(s) ≤5 years	U	0.31	0.43	-26.3		-5.28	0.00	.
	M	0.31	0.31	1.10	95.9	0.23	0.82	.
Mother is working	U	0.34	0.27	15.5		3.09	0.00	.
	M	0.34	0.35	-1.40	90.9	-0.29	0.78	.
Primary education	U	0.61	0.66	-9.10		-1.82	0.07	.
	M	0.62	0.61	0.90	89.9	0.19	0.85	.
Secondary education	U	0.03	0.07	-18.2		-3.70	0.00	.
	M	0.03	0.02	2.00	89.2	0.54	0.59	.
Tertiary education	U	0.21	0.19	4.90		0.97	0.33	.
	M	0.21	0.22	-1.00	79.7	-0.20	0.84	.
2nd Wealth quintile	U	0.21	0.21	-1.60		-0.31	0.75	.
	M	0.21	0.21	0.30	79.8	0.07	0.95	.
3rd Wealth quintile	U	0.13	0.19	-16.8		-3.39	0.00	.
	M	0.13	0.13	0.70	95.9	0.16	0.88	.
4th Wealth quintile	U	0.15	0.19	-10.2		-2.06	0.04	.
	M	0.15	0.15	-1.50	85.1	-0.33	0.74	.
5th Wealth quintile	U	0.57	0.47	18.6		3.72	0.00	.
	M	0.56	0.57	-1.40	92.5	-0.29	0.77	.
Urban	U	0.28	0.39	-25.0		-5.02	0.00	.
	M	0.28	0.27	2.30	90.7	0.51	0.61	.
>50% forest/barren land	U	0.30	0.33	-7.10		-1.43	0.15	.
	M	0.30	0.30	-0.20	97.5	-0.04	0.97	.
>50% grass and wood land	U	0.43	0.36	13.7		2.73	0.01	.
	M	0.43	0.44	-0.90	93.1	-0.19	0.85	.
>50% built up land	U	0.12	0.17	-12.6		-2.54	0.01	.
	M	0.12	0.13	-2.20	82.4	-0.49	0.63	.
land cover associations	U	0.11	0.12	-3.70		-0.74	0.46	.
	M	0.11	0.09	4.60	-22.8	0.99	0.32	.
LGP[a]: 76-120 days	U	0.18	0.17	0.40		0.07	0.94	.
	M	0.17	0.17	0.10	70.7	0.02	0.98	.
LGP[a]: 121-180 days	U	0.66	0.69	-6.10		-1.23	0.22	.
	M	0.67	0.66	0.40	93.7	0.08	0.94	.
LGP[a]: >180 days	U	0.10	0.09	0.60		0.11	0.91	.
	M	0.10	0.10	-2.00	-261.4	-0.41	0.68	.
Farming system 1[b]	U	0.01	0.01	4.20		0.83	0.41	.
	M	0.01	0.01	-2.10	50.2	-0.39	0.70	.
Farming system 2[c]	U	0.70	0.73	-6.30		-1.26	0.21	.
	M	0.70	0.72	-4.50	29.2	-0.93	0.35	.
Farming system 3[d]	U	0.01	0.01	8.30		1.63	0.10	.
	M	0.01	0.01	-3.10	62.4	-0.58	0.56	.
Farming system 4[e]	U	0.20	0.18	6.20		1.24	0.21	.
	M	0.20	0.19	4.20	33.1	0.86	0.39	.
Slope 8-30 degrees	U	0.68	0.70	-5.10		-1.02	0.31	.
	M	0.68	0.69	-2.20	57.7	-0.45	0.65	.
Slope >30 degrees	U	0.10	0.11	-4.30		-0.87	0.38	.
	M	0.10	0.11	-1.80	58.3	-0.38	0.70	.

[*] If variance ratio outside [0.88; 1.14] for U and [0.88; 1.14] for M. [a] LGP= length of growing period. [b] Root crop/Cereal-root crop mixed. [c] Maize mixed. [d] Large commercial and smallholder/Pastor. [e] Agropastoral millet sorghum.

References

Austin, P. (2009): "Balance diagnostics for comparing the distribution of baseline covariates between treatment groups in propensity-score matched samples", *Statistics in Medicine*, 28, 3083-107.

Rosenbaum, P. And D. Rubin (1985): "Constructing a Control Group Using Multivariate Matched Sampling Methods That Incorporate the Propensity Score", *The American Statistician*, 39(1), pp.33-38.

Stuart, E., B. Lee, and F. Leacy (2013): "Prognostic score–based balance measures can be a useful diagnostic for propensity score methods in comparative effectiveness research", *Journal of Clinical Epidemiology*, 66, S84-90.
