# Supplementary material for: Who lacks and who benefits from diet diversity: evidence from (impact) profiling for children in Zimbabwe
Source: Int J Health Geogr. 2020 Nov 4;19:45. doi: 10.1186/s12942-020-00240-2 (PMC7640455; doi:10.1186/s12942-020-00240-2)
Supplement: Supplementary file 5 — Additional file 5: Signs of estimated ATT effects and trade-off minimum profile sample size versus FDR. [file 12942_2020_240_MOESM5_ESM.pdf]

Additional File 5: Signs of estimated ATT effects and trade-off minimum profile sample size versus FDR

Figure S.5.1. Number of profiles with positive/negative estimated ATT and (absolute) t-value≥1.645

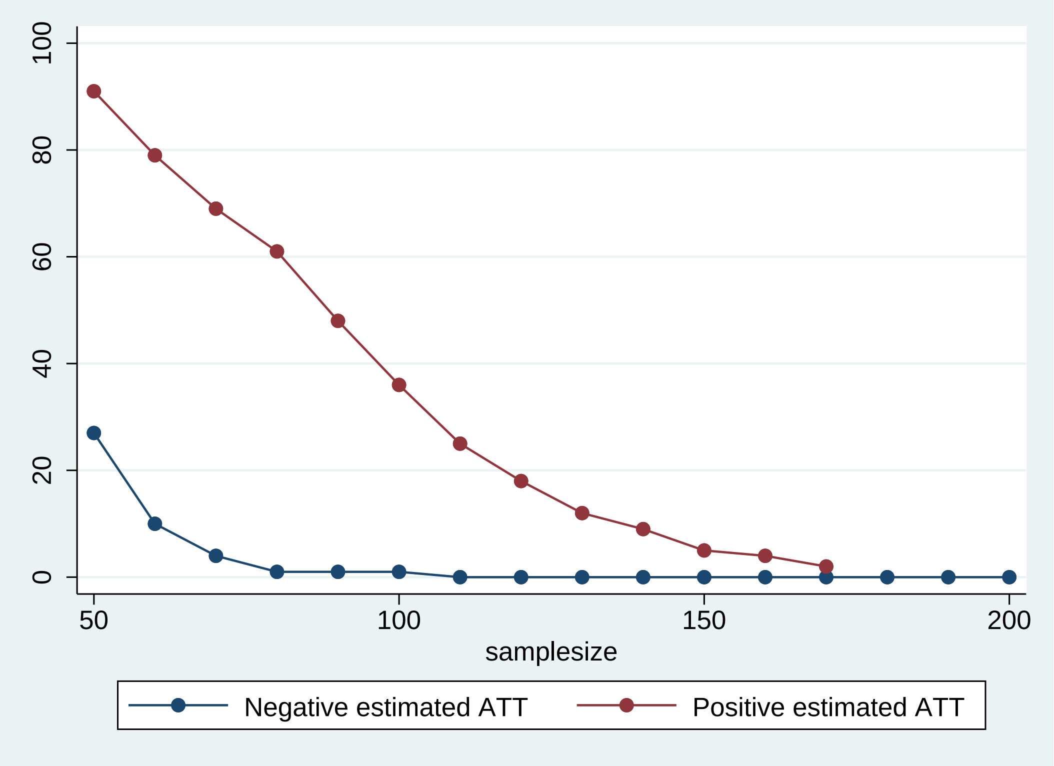

Due to the presence of sampling variation, we need to decide which profiles we want to consider. Here we face a trade-off in terms of the minimum profile sample size and the false discovery rate (FDR) as limiting the analysis to profiles with larger sample sizes reduces the false discovery rate but also the available number of profiles. Table S.5.1 illustrates this trade-off for profiles with a t-value of 1.645 or higher (5% significance in one-sided test). Increasing the minimum profile sample size from 50 to 150, reduces the number of profiles from 671 to 22. The FDR is given by the expected number of profiles assuming that ATT=0 divided by the actual number of statistically significant profiles (t-value of 1.645 or higher), and this rate is falling as well, from 0.37 to 0.14.[1]

[1] The FDR does not fall monotonically throughout because of small sample variation when the minimum sample size becomes large.

Table S.5.1. FDR and total number of profiles discovered by minimum profile sample size

Minimum profile sample size	Number of profiles present	Randomly expected number of profiles (ATT=0, t≥ 1.645)	Actual number of significant profiles (t≥ 1.645)	False discovery rate (FDR)
50	671	33.6	91	0.37
75	257	12.9	65	0.20
100	115	5.8	36	0.16
125	45	2.3	16	0.14
150	22	1.1	5	0.22

Because the FDR is only slightly lower at even higher minimum sample sizes, we focus on the 36 profiles with at least 100 observations and an ATT with t-value of 1.645 or higher.[2]

[2] Note that we focus only on profiles with a positive estimated ATT effect, which is justified by our clear evidence (as well as theory and prior evidence) that the impact of diet diversity is nonnegative.
