# Supplementary material for: Who lacks and who benefits from diet diversity: evidence from (impact) profiling for children in Zimbabwe
Source: Int J Health Geogr. 2020 Nov 4;19:45. doi: 10.1186/s12942-020-00240-2 (PMC7640455; doi:10.1186/s12942-020-00240-2)
Supplement: Supplementary file 6 — Additional file 6: Winning profiles in (impact) profiling. [file 12942_2020_240_MOESM6_ESM.pdf]

Additional File 6: Winning profiles in (impact) profiling

Polling approach

Table S.6.1. presents the winning profiles using the polling approach.

Table S.6.1. Polling approach: Winning profile details and statistics

	$0 \leq \alpha \leq 0.7$	$0.8 \leq \alpha \leq 1$
Child characteristics		
Age of child		6-12 Months -
Household characteristics		
Wealth tercile	Poorest/poor	
Education	Secondary	
Working status mother	Mother does not work	
Environmental variables		
Location	Rural	Rural
Slope		8-30 Degrees
Length growing period	121-180 Days	121-180 Days
Land use	>50% grass and woodland	
Farming system		Maize/mixed system
Exclusion error $\lambda_{polling}$	0.96	0.77
Inclusion error $\mu_{polling}$	0.09	0.21
Indicator $\psi_{polling}$	0.09-0.48	0.59-0.77

Source: own calculations based on DHS and geospatial information.

Two observations are in order here. First, for all profiles, the exclusion error is quite large if only the “winning” households are targeted, with the majority of the households suffering from insufficient diet diversity being excluded. This can be remedied by targeting households characterized by profiles, say, in the top-5 of profiles, but an alternative approach could be to set an upper bound on the exclusion error and subsequently adjust the number of variables to be included in a profile. This would obviously also increase the inclusion error, and a new analysis would have to be carried out to find optimal profiles.

Second, the selection of the winning profiles is based on the entire sample, which could imply large regional differences in inclusion and exclusion errors. We have checked and found spatial diversity in inclusion and exclusion errors for given winning profiles suggesting a need to determine province-specific profiles. However, the limited number of observations does not allow for such a spatially

explicit analysis but the inclusion of spatially explicit information on natural resource distributions partly compensates for this.

Impact profiling approach

Table S.6.2 presents the “winning” impact profiles among the 36 profiles that minimize the weighted geometric average for different values of α. There are three different winning profiles in total. We note the following. First, there is a partial but certainly not perfect overlap in the winning polling and impact profiles (cf. Tables S.6.1 and S.6.2). This suggests that profiling on need can substitute to some extent for profiling on impact, but not entirely. Second, Table S.6.2 suggests a pro-poor policy among non-working mothers in rural areas with a medium length of growing period, maize/mixed farming systems and moderate slopes. Third, a focus on children of 6-12 months old is warranted if minimizing the impact exclusion error is especially desired (large α), while a focus on households with multiple young children is especially justified when minimizing the impact inclusion error (small α).

Table S.6.2. Impact profiling approach: Winning impact profile details and statistics

	$0 \leq \alpha < 0.56$	$0.56 \leq \alpha < 0.71$	$0.71 \leq \alpha \leq 1$
Child characteristics			
Age of child	-	-	6-12 Months
Has sibling(s) of ≤5 years	Yes	Yes	-
Household characteristics			
Wealth tercile	Poorest/poorer	Poorest/poorer	-
Working status of mother	Not working	Not working	-
Environmental			
Location	Rural	Rural	Rural
Slope	8-30 Degrees	-	8-30 Degrees
Length growing period	121-180 Days	121-180 Days	121-180 Days
Farming system	-	Maize/mixed system	Maize/mixed system
Exclusion error λ_{impact}	0.79	0.78	0.61
Inclusion error μ_{impact}	0.29	0.31	0.53
Indicator ψ_{impact}	0.30-0.51	0.52-0.59	0.59-0.62

Source: own calculations based on DHS (2015) and geospatial information
